# Supplementary material for: A Divergent Articulavirus in an Australian Gecko Identified Using Meta-Transcriptomics and Protein Structure Comparisons
Source: Viruses. 2020 Jun 4;12(6):613. doi: 10.3390/v12060613 (PMC7354609; doi:10.3390/v12060613)
Supplement: Supplementary file 1 [file viruses-12-00613-s001.zip › viruses-807067.suppl zip/Ortiz.Figure S1.pdf]

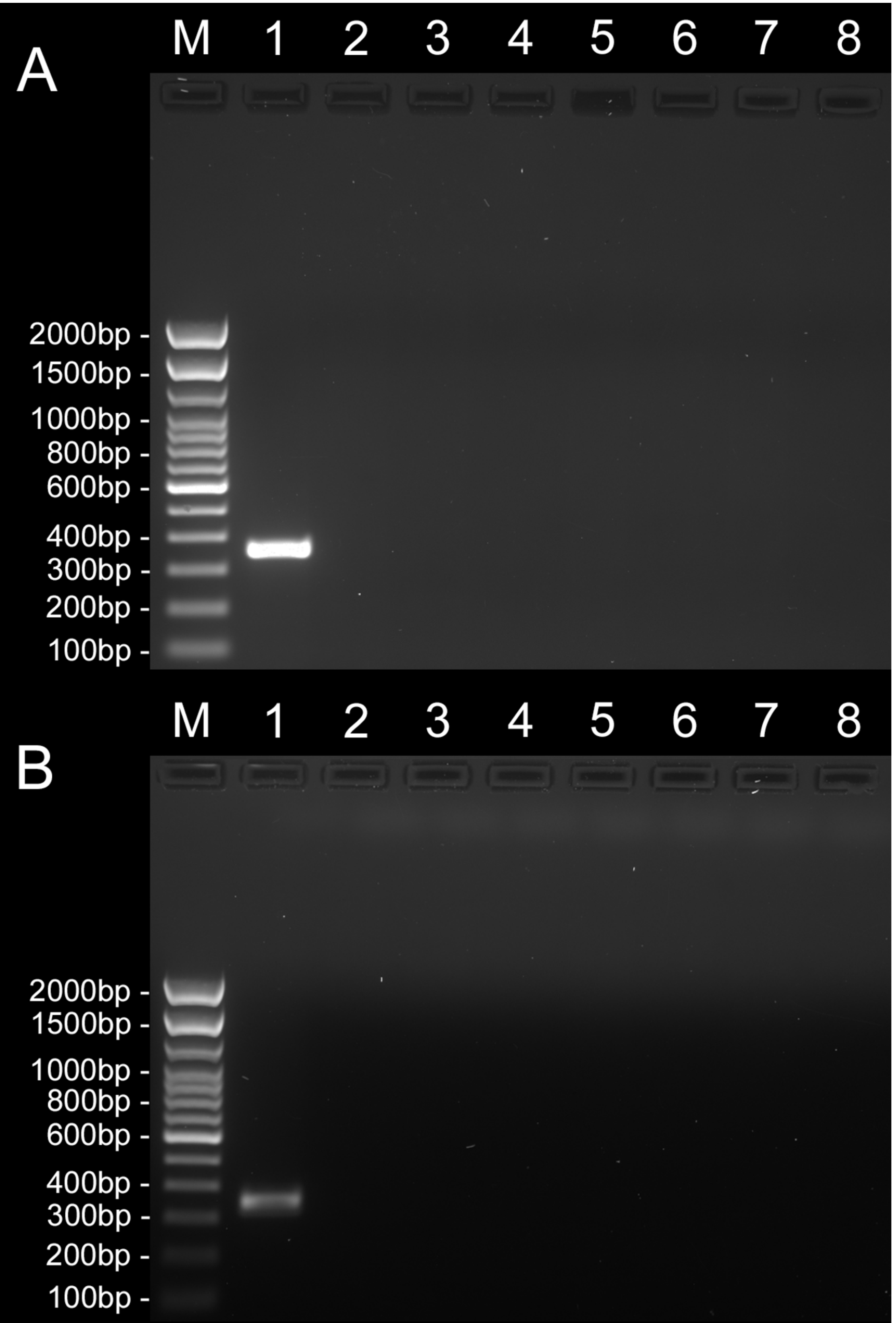

C

ROW	LABEL	SAMPLE	PCR	TARGET	HOST	VOL (µl)
A	M	100bp DNA ladder	~	~	~	9
	1	CCM0247 Liver	F2V7	Ortho	*Gehyra lauta*	6
	2	CCM0294 Liver	F2V7	Ortho	*Carlia amax*	6
	3	CCM0485 Liver	F2V7	Ortho	*Heteronotia binoei*	6
	4	CCM0533 Liver	F2V7	Ortho	*Gehyra nana*	6
	5	CCM0732 Liver	F2V7	Ortho	*Carlia gracilis*	6
	6	CCM0736 Liver	F2V7	Ortho	*Carlia munda*	6
	7	CCM1801 Liver	F2V7	Ortho	*Heteronotia planiceps*	6
	8	Water	F2V7	Ortho	~	6
B	M	100bp DNA ladder	~	~	~	9
	1	CCM0247 Liver	F3V7	Ortho	*Gehyra lauta*	6
	2	CCM0294 Liver	F3V7	Ortho	*Carlia amax*	6
	3	CCM0485 Liver	F3V7	Ortho	*Heteronotia binoei*	6
	4	CCM0533 Liver	F3V7	Ortho	*Gehyra nana*	6
	5	CCM0732 Liver	F3V7	Ortho	*Carlia gracilis*	6
	6	CCM0736 Liver	F3V7	Ortho	*Carlia munda*	6
	7	CCM1801 Liver	F3V7	Ortho	*Heteronotia planiceps*	6
	8	Water	F3V7	Ortho	~	6
