# Supplementary material for: A Divergent Articulavirus in an Australian Gecko Identified Using Meta-Transcriptomics and Protein Structure Comparisons
Source: Viruses. 2020 Jun 4;12(6):613. doi: 10.3390/v12060613 (PMC7354609; doi:10.3390/v12060613)
Supplement: Supplementary file 1 [file viruses-12-00613-s001.zip › viruses-807067.suppl zip/Ortiz.Supplementary Material.pdf]

Supplementary Materials

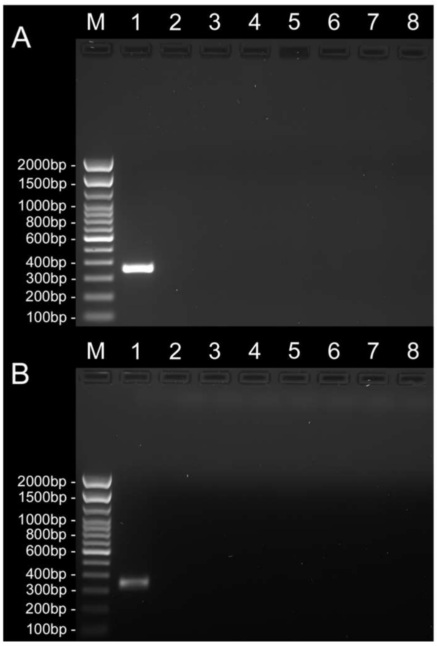

c

ROW	LABEL	SAMPLE	PCR	TARGET	HOST	VOL (μl)
A	M	100bp DNA ladder	~	~	~	9
	1	CCM0247 Liver	F2V7	Ortho	*Gehyra lauta*	6
	2	CCM0294 Liver	F2V7	Ortho	*Carlia amax*	6
	3	CCM0485 Liver	F2V7	Ortho	*Heteronotia binoei*	6
	4	CCM0533 Liver	F2V7	Ortho	*Gehyra nana*	6
	5	CCM0732 Liver	F2V7	Ortho	*Carlia gracilis*	6
	6	CCM0736 Liver	F2V7	Ortho	*Carlia munda*	6
	7	CCM1801 Liver	F2V7	Ortho	*Heteronotia planiceps*	6
	8	Water	F2V7	Ortho	~	6
B	M	100bp DNA ladder	~	~	~	9
	1	CCM0247 Liver	F3V7	Ortho	*Gehyra lauta*	6
	2	CCM0294 Liver	F3V7	Ortho	*Carlia amax*	6
	3	CCM0485 Liver	F3V7	Ortho	*Heteronotia binoei*	6
	4	CCM0533 Liver	F3V7	Ortho	*Gehyra nana*	6
	5	CCM0732 Liver	F3V7	Ortho	*Carlia gracilis*	6
	6	CCM0736 Liver	F3V7	Ortho	*Carlia munda*	6
	7	CCM1801 Liver	F3V7	Ortho	*Heteronotia planiceps*	6
	8	Water	F3V7	Ortho	~	6

Figure S1. PCR detection and host association of *Lauta virus*. (**a-b**) Agarose gels electrophoresis showing PCR products from two sets of primers that target a region in the PB1 gene segment (RdRp). Samples correspond to (**c**) liver tissue from seven different reptile species. A 355 bp PCR product was only amplified in *G. lauta.*

Table S1. Summary of the contig alignment to genomic segments of TiLV using DIAMOND. The relative abundance of each transcript was also calculated (see Methods).

Table S2. Summary of hits recovered after alignment of the untranslated contigs with reference protein sequences of the RdRp subunit PB1. The custom database included virus reference sequences from the order *Articulavirales*.

Table S3. List of virus sequences used in the phylogenetic analysis. All sequences correspond to the PB1 protein.

Species name	Acronym	GenBank access code	Family
Blueberry mosaic associated virus	BlMaV	YP_009449565	*Ophioviridae* - Outgroup
Montano orthohantavirus	MTNV	YP_009361849	*Hantaviridae* - Outgroup
Bayou orthohantavirus	BAYV	YP_009505596	*Hantaviridae* - Outgroup
Influenza A virus	FLUAV	YP_009118628	*Orthomyxoviridae*
Influenza B virus	FLUBV	NP_056657	*Orthomyxoviridae*
Influenza D virus	FLUDV	YP_009449556	*Orthomyxoviridae*
Influenza C virus	FLUCV	YP_089653	*Orthomyxoviridae*
Salmon isavirus	ISAV	YP_145804	*Orthomyxoviridae*
Quaranfil quaranjavirus	QRFV	YP_009508043	*Orthomyxoviridae*

Thogoto thogotovirus	THOV	YP_145794	*Orthomyxoviridae*
Tilapia tilapinevirus	TiLV	YP_009246481	*Amnoonviridae*
Dhori thogotovirus	DHOV	YP_009352882	*Orthomyxoviridae*
Oz virus	OZV	YP_009553280	*Orthomyxoviridae*
Wellfleet Bay virus	WFBV	YP_009110686	*Orthomyxoviridae*
Johnston Atoll quaranjavirus	JAV	YP_009665204	*Orthomyxoviridae*

Table S4. Set of primers used for PCR and Sanger sequencing reactions.

Primer	Nucleotide sequence (5′-3′)	Tm (ºC)	Anneal (ºC)	Amplicon size (bp)
F2V7_136F	ACTGCACCAAGTTCAACGGA	61.8	65.2	355
F2V7_490R	GTGAGTGGGTCCATCTTGCA	61.7		
F3V7_396F	TGCAGTAGAGCGGAGGTAGA	61.6	64.4	355
F3V7_711R	ATCCGAGCCCAGCATATCTC	60.9		
